# Role Modeling Kindness at the Bedside

**DOI:** 10.7759/cureus.57078

**Published:** 2024-03-27

**Authors:** Lauren Fine, Tina Takla, Vijay Rajput

**Affiliations:** 1 Dr. Kiran C. Patel College of Allopathic Medicine, Nova Southeastern University, Fort Lauderdale, USA; 2 Dr. Kiran C. Patel College of Osteopathic Medicine, Nova Southeastern University, Fort Lauderdale, USA

**Keywords:** role modeling, generations, compassion, bedside teaching, empathy, kindness

## Abstract

Compassion and kindness are interchangeable attitudes and behaviors in society. As evidence shows the importance of compassion and kindness in healthcare, there has been a push to nurture and teach compassion through experiential learning in medical schools. However, there is not much evidence of educating learners on the importance of kindness as the complement or foundation of compassion and empathy. Kindness is the ability to act positively and appropriately and can be provided without emotion, judgment, or expecting anything in return. Kindness does not require the receiver to be in severe distress or suffering. Acts of kindness can be random acts done to anyone, anytime, with or without illness or suffering. Research shows that kindness elevates the healthcare profession for both clinicians and patients. Compassion and kindness must be taught through the integrated approach of role modeling, observation, practice, experience, and reflection in the classroom and in the clinical environment. It is vital that medical schools and healthcare institutions’ faculty and staff make kindness to patients, families, and staff a key behavior, along with compassion and empathy. There is more that can be done to encourage acts of kindness through everyday actions; educators can display kindness toward colleagues and medical students in their learning. Kindness can improve conversations with patients and improve the emotional and social well-being of learners. Displaying kindness during bedside or classroom teaching would engrain its importance in the professional identity formation of future generations of physicians.

## Editorial

“I need money to get a bus home,” one of my patients from Camden, New Jersey, pleaded with me once. My residents and students were hesitant, if not opposed, to giving the patient money since he had a long history of substance use disorder. They strongly believed that giving the patient money would encourage him to spend the money on drugs. I thought it was better to give him money for the bus home because it may give him more of what he needs to stop using drugs, and not giving him money would discourage him from asking for help at this vulnerable moment. There can be some concern when it comes to spreading random acts of kindness or generosity, that the recipient will misuse that kindness. However, that is usually far from the case. Throughout my life and career, I have had the habit of giving smaller amounts of money to those who asked me.

There were several times my patients did not have money to buy food because they would run out of money before their next social security check arrived. My patients would typically ask for money for groceries. “Can you survive with 20 dollars per week?,” I asked my residents and students, who usually spend the same amount of money on their morning coffee or happy hour on Friday evenings.

“One loaf of bread, one gallon of milk, a dozen bananas, 12 eggs, a jar of peanut butter, and a jar of jelly can give you enough calories to survive for the week,” I would tell my residents and students. I give my patients money as well as a list of foods they should buy. The question is asked repeatedly whether this type of kindness or generosity is beneficial or whether my students and residents are correct that patients will go and spend that money on drugs and alcohol.

As professionals in medicine, we are all very well trained in showing compassion to patients and families for their illnesses, distress, and sometimes the sudden sad news we have to deliver. Can these random acts of generosity during bedside teaching foster more random acts of kindness among our learners? Many of my students want to make a difference in the community and at a global level. They are compassionate and altruistic in their behavior. However, are they missing opportunities to make a difference, one patient at a time, at the expense of a desire to make an impact on a larger scale?

There was a time when I spent two to three weeks at a time in the inpatient hospital teaching service. Every six months to a year, we get a patient whose birthday falls during their hospitalization. I would always make sure that I gave money to my senior resident or student to pick up a small cake on their way to work the next day. If a patient is in a position to celebrate their birthday, the team, including faculty, residents, nurses, and occasionally family members, would sing a birthday song with a small cake adorned with a single candle to ensure their birthday was celebrated, even in the middle of their illness.

Given the current state of the learning environment, asking students to get a cake for a patient may be perceived as a waste of their time and even an abuse of the learning environment. However, I do not recall any of my students or healthcare team members expressing that this type of random kindness was a waste of time or otherwise conveying negative messages about it. Learners identified caring as one of the key components they needed more of in their classroom from their teachers. When teachers showed they cared and incorporated compassion, or, in our case, kindness, into their teaching strategies, students felt more invested in the class. It also makes a teacher more approachable when they are observed demonstrating understanding and kindness toward patients. It is almost too simple to say that incorporating kindness into care is positively associated with students’ care for their own academic improvement, but that is what we see happening [1].

Employing a nonjudgmental approach toward this kindness can enhance bidirectional conversation, thus requiring the learner to take more initiative for patient advocacy. It is evident that current standards in medical education do not strongly emphasize the importance of nonjudgmental conversation along with random acts of kindness. The concept of “three-way nonjudgmental listening with random acts of kindness and generosity” can give learners the opportunity to see the value of kindness while simultaneously promoting mutual respect.

Active and open nonjudgmental listening, gratitude, and kindness can maximize the bedside experience and enhance the conversation between learner, patient, and teacher. The use of kindness and random generosity at the bedside can help learners feel more fulfilled, boost their self-esteem, improve their self-evaluation, and trigger positive emotions. Learners who have a more positive response to kindness are more likely to embrace a growth mindset regarding the random act of kindness and thereby focus on their future patient-physician relationships.

Compassion, empathy, and kindness seemingly overlap and are often used interchangeably. Empathy and compassion are distinguished based on the intensity of suffering and the response of others to that suffering through actions or emotions. In contrast, kindness is a positive and appropriate benevolent act without emotional attachment. It demonstrates sensitivity to the needs, thoughts, and feelings of others but does not require one to show vulnerability, distress, or suffering [2].

Compassion and empathy encompass the basis for humanistic, patient-centered care. Currently, there is a crisis of burnout among medical students and compassion-fatigued physicians. A steep decline in empathy occurs with the greatest frequency in the clinical years of medical school. Psychological protections to decrease burnout and emotional distress may result in medical students dissociating from patients and creating barriers that diminish kindness in patient-doctor interactions [3].

To counter this alarming trend, the authors introduce the concept of teaching kindness, which is novel to medical education and professional development. Kindness is a less complex concept to teach compared to empathy due to the nature and context surrounding empathy. Practicing empathetic care requires medical students to fully invest in patients and acquire patient perspectives, which is more difficult than implementing simple acts and gestures of kindness.

Physicians who show kindness to patients allow for the creation of a trusting and caring environment. This can be done through even the smallest actions or microexpressions, such as smiling, actively listening, or asking about the patient’s life. Small acts of kindness take little to no time to implement and have a major positive impact on the patient-doctor relationship and the treatment plan. Studies have shown that kindness in medicine has allowed for better health outcomes, greater patient adherence to care plans, and increased communication [4].

Research has demonstrated the positive impact of incorporating kindness into the practice of medicine. There is also data that indicates that acts of kindness may be decreasing due to generational changes that impact social development, which is negatively impacting patient-physician interactions. Concluding remarks demonstrate the importance of integrating kindness into medical school curricula.

Multiple experiments have demonstrated that random acts of kindness and prosocial actions have a positive effect on the well-being of both the receiver and the giver [5]. There is a need to cultivate random acts of kindness among students toward patients, peers, and the public without expecting anything in return. There should be an emphasis on the delivery of kindness without a sense of obligation.

It is critical that the research and practice of kindness be integrated into the medical school curricula and in continuing education for physicians. Furthermore, as teachers and mentors for students, we should role model kindness through amiability, congeniality, and respect in our everyday interactions with learners and others.
